# The impact of active case finding on transmission dynamics of tuberculosis: A modelling study

**DOI:** 10.1371/journal.pone.0257242

**Published:** 2021-11-19

**Authors:** Diepreye Victoria Ayabina, M. Gabriela M. Gomes, Nhung Viet Nguyen, Luan Vo, Suvesh Shreshta, Anil Thapa, Andrew James Codlin, Gokul Mishra, Maxine Caws

**Affiliations:** 1 Liverpool School of Tropical Medicine, Liverpool, United Kingdom; 2 Big Data Institute, Li Ka Shing Centre for Health Information and Discovery, University of Oxford, Oxford, United Kingdom; 3 CIBIO-InBIO, Centro de Investiga¸c˜ao em Biodiversidade e Recursos Gen´eticos, and CMUP, Centro de Matem´atica da Universidade do Porto, Porto, Portugal; 4 National Tuberculosis Control Programme of Vietnam- National Lung Hospital (VNTP-NLH), Hanoi, Vietnam; 5 Friends for International TB Relief (FIT), Ho Chi Minh City, Vietnam; 6 Save the Children, Kathmandu, Nepal; 7 National TB Control Centre, Thimi, Kathmandu, Nepal; 8 Birat Nepal Medical Trust, Kathmandu, Nepal; Texas A&M University College Station, UNITED STATES

## Abstract

**Background:**

In the last decade, active case finding (ACF) strategies for tuberculosis (TB) have been implemented in many diverse settings, with some showing large increases in case detection and reporting at the sub-national level. There have also been several studies which seek to provide evidence for the benefits of ACF to individuals and communities in the broader context. However, there remains no quantification of the impact of ACF with regards to reducing the burden of transmission. We sought to address this knowledge gap and quantify the potential impact of active case finding on reducing transmission of TB at the national scale and further, to determine the intensification of intervention efforts required to bring the reproduction number (*R*_0_) below 1 for TB.

**Methods:**

We adopt a dynamic transmission model that incorporates heterogeneity in risk to TB to assess the impact of an ACF programme (IMPACT TB) on reducing TB incidence in Vietnam and Nepal. We fit the models to country-level incidence data using a Bayesian Markov Chain Monte Carlo approach. We assess the impact of ACF using a parameter in our model, which we term the treatment success rate. Using programmatic data, we estimate how much this parameter has increased as a result of IMPACT TB in the implementation districts of Vietnam and Nepal and quantify additional efforts needed to eliminate transmission of TB in these countries by 2035.

**Results:**

Extending the IMPACT TB programme to national coverage would lead to moderate decreases in TB incidence and would not be enough to interrupt transmission by 2035. Decreasing transmission sufficiently to bring the reproduction number (R0) below 1, would require a further intensification of current efforts, even at the sub-national level.

**Conclusions:**

Active case finding programmes are effective in reducing TB in the short term. However, interruption of transmission in high-burden countries, like Vietnam and Nepal, will require comprehensive incremental efforts. Complementary measures to reduce progression from infection to disease, and reactivation of latent infection, are needed to meet the WHO End TB incidence targets.

## Introduction

Tuberculosis (TB) is ranked as one of the top ten causes of global mortality and morbidity, responsible for an estimated 1.7 million deaths per year. The global incidence is only declining gradually, at 1 − 2% per year and it is unlikely that the ‘end’ of TB as an epidemic and major public health problem forthcoming. Even though TB is an ancient disease with effective preventative and curative treatment, it is estimated that worldwide 10 million people develop active TB disease each year [1]. To make headway and meet the targets of the End TB strategy set by the World Health Organization (WHO) towards achieving elimination of TB [2], there is need for a deeper understanding of the interaction between TB transmission dynamics and the different prevention and care strategies.

The basic reproduction number, *R*_0_, which estimates the speed at which an infectious disease can spread in a population is a key quantity in describing the fate of an epidemic. It is described as the average number of secondary cases caused by a primary infectious case in an entirely susceptible population. The reproduction number depends on the probability of transmission for an infectious contact, the probability of progressing to active disease after infection and the duration of infectiousness. Interventions that directly influence one or a combination of these processes will modify the reproduction number and thereby affect disease transmission [3].

Tuberculosis is preventable and curable, and its transmission can be prevented by prompt identification and treatment of infectious individuals. Case finding strategies that rely solely on passive presentation of people with active TB disease to the health system may not be enough to interrupt transmission dynamics [3]. Progress has been made in closing the notification gap in some high burden countries, with an 8.5% increase in the global TB notification from the previous year in 2018, which was driven largely by increasing notification from the private sector. However, there remains an estimated gap of 3 million between the estimated number of incident cases and the number of new cases reported [1]. Intensified efforts such as active case finding (ACF) programmes are required, especially in high TB burden countries to improve access and utilization of TB diagnostic and treatment services.

Over the last decade, active case finding strategies for TB have been implemented in many diverse settings. Among these were twenty-eight case finding projects facilitated by the TB REACH programme (sponsored by the Canadian government) [4]. Results from these case detection projects describe a diversity of interventions in a variety of settings with some showing a large increase in the number of people treated for TB. However, the evidence base to inform policy development for national ACF scale-up strategies remains limited. In particular, policy makers have limited evidence of cost-effectiveness and long-term impact on the overall epidemic trajectory from existing data. The IMPACT TB programme (European Union Horizon 2020 grant 733174) aimed to address some of the knowledge gaps by implementing ACF activities targeted at high risk individuals at the district-level (six districts of Ho Chi Minh City, Vietnam, and four districts in Nepal; covering approximately 2.6 million population in each country) while systematically evaluating aspects including facilitators, barriers and health economics of the scale up of ACF in these countries. The goal of the project is supported by two main strategies in each country: salaried employees versus volunteer counsellors in Vietnam, and traditional smear microscopy versus Xpert Omni molecular testing in Vietnam. More details of these can be found in [5–13]. Both countries have a high incidence of TB. Vietnam is a middle-income country with a strong, centralized National TB Programme, while Nepal is one of the world’s least developed countries with a fragmented and weak health infrastructure.

For many infectious diseases, including TB, it is well known that the risk of disease is increased among some population sub-groups. Risk of TB can be viewed as a three-dimensional phenomenon: individual characteristics that increase exposure or susceptibility to infection, progression to disease after infection, and duration of infectiousness. These various forms of risk contribute to the heterogeneous distribution of the global burden across populations. Trauer et al [14] in a recent publication, provide a conceptual framework for understanding heterogeneity in TB epidemiology and point out that the drivers of variation in TB burden are diverse and include characteristics of the infecting organism, infectious host, susceptible host, environment and social determinants. Although the risk factors associated with each of these drivers may differ considerably, they interact to reduce or amplify heterogeneity. To include these various forms of risk in a transmission model for TB, a straightforward procedure would be to divide the population according to the risk factors of interest and consider the natural history of TB in each of these groups. Besides being a formidable task, this approach would ignore many relevant factors and under-represent the full extent of heterogeneity present in any study population, which could undermine the ability of models to predict the impact of interventions. An alternative is to estimate distributions of unobserved risk of TB by using case notification data and then incorporate these distributions in a mathematical model of TB transmission [15].

There have been several studies which seek to provide evidence for the benefits of ACF to communities and individuals. The review by Kranzer et al [16] provides a comprehensive summary of potential beneficial effects of ACF with a focus on four specific questions relating to the proportion of cases found, duration of infectiousness, and treatment outcomes and TB incidence. The review stresses that there is much uncertainty about the impact of earlier diagnosis on patient outcomes and transmission. Two more recent studies [17,18] show that ACF is more effective in enhancing earlier diagnosis and averting TB cases. However, there remains no quantification of the impact of ACF with regards to reducing transmission and hence the value of *R*_0_. Here, we sought to theoretically quantify the potential impact of ACF on reducing country level burden of transmission of TB. Using a dynamic model of TB transmission that incorporates heterogeneity of risk, we investigate the conditions for reducing TB transmission in Nepal and Vietnam between 2017 and 2020: the period during which the IMPACT TB programme was implemented in these countries.

## Methods

### Model structure

To analyse the dynamics of TB epidemiology, we adopt a TB transmission model from previous studies [15,19–21]. We represent heterogeneity in terms of contact rates as in [15]. We consider that average contact rates vary among individuals in a population, potentially according to some continuous distribution which we discretize into two risk groups: low and high risk groups with proportions *p*_1_ and *p*_2_, respectively. Within each group, individuals are classified, according to their infection history, into uninfected (*U*_*i*_) or infected in one of three possible states: primary infection (*P*_*i*_); latent infection (*L*_*i*_); and active TB disease (*I*_*i*_), which is the infectious state.


$$\begin{array}{l} \frac{dU_{i}}{dt}=p_{i}\mu +\theta \tau I_{i}-\lambda_{i}U_{i}-\mu U_{i}\\ \frac{dP_{i}}{dt}=\lambda_{i}(U_{i}+L_{i})-(\delta +\mu )P_{i}\\ \frac{dI_{i}}{dt}={\phi \delta P}_{i}+\omega L_{i}-(\tau +\mu )I_{i}\\ \frac{dL_{i}}{dt}=(1-\phi )\delta P_{i}+(1-\theta )\tau I_{i}-\lambda_{i}L_{i}-(\omega +\mu )L_{i}, \end{array}$$ (1)

The model parameters along with typical values used herein are listed in Table 1. The force of infection, *λ*_*i*_, acting on each risk group is

$$\lambda_{i}=\frac{\alpha_{i}}{\langle \alpha \rangle }\beta (\alpha_{1}I_{1}+\alpha_{2}I_{2}),$$ (2)

where *α*_*i*_ is the risk of individuals in group *i* in relation to the population mean 〈*α*〉 = 1, and the basic reproduction number is

$$R_{0}=\frac{\langle \alpha^{2}\rangle }{\langle \alpha \rangle }[\frac{\omega +\mu }{\mu (\tau +\omega +\mu )+\theta \tau \omega }][\frac{\phi \delta }{\delta +\mu }+\frac{(1-\phi )\delta \omega }{\left(\delta +\mu )(\omega +\mu \right)}]\beta ,$$ (3)

Where 〈*α*^2^〉 is the second moment of the risk distribution [15].

**Table 1:** Parameters for tuberculosis transmission model.

Symbol	Definition	Value
*β*	Mean effective contact rate	varying (*yr*^−1^)
*μ*	Death and birth rate	1/80 *yr*^−1^
*δ*	Rate of progression from primary infection	2 *yr*^−1^
*ϕ*	Proportion progressing from primary to active TB	varying
*ω*	Rate of reactivation of latent infection	varying (*yr*^−1^)
*τ*	Rate of successful treatment	varying (*yr*^−1^)
*θ*	Proportion clearing infection upon treatment	0; 1
*σ*	Factor affecting susceptibility due to previous infection	1; 0.5
*α*_*i*_	Individual risk in relation to population average	estimated
*p*_*i*_	Proportion of individuals in low and high-risk groups	0.96; 0.04

### Model initialization

To initialize the model, we simulated a virgin epidemic and run to equilibrium, which was assumed to represent the starting conditions in 2002 in Vietnam and 2009 in Nepal. Based on WHO data, prior to these years the incidence trends for Vietnam and Nepal did not change significantly. Instead of estimating the variance of the risk distribution as in [15], we use variance as a sensitivity parameter in our analysis with values in the set *var*(*α*) = (5,10,15). The proportion of the population in the risk groups are set at *p*_1_ = 0.96 and *p*_2_ = 0.04. Birth and death rates are assumed equal and set to 1/80 per year for both countries.

### Intervention strategies

The model incorporates different control interventions that can be implemented to reduce TB incidence. We consider three approaches which can be implemented individually or in combination. Declines in TB incidence can be attributed to a reduction in progression (reducing the parameter *ϕ*), reducing reactivation (reducing the parameter *ω*), and increasing the treatment success rate (increasing the parameter *τ*). We refer to the first two as preventive interventions, as these reduce the rate with which individuals develop active TB, by either slowing the rate of progression from infection or by shrinking the reservoir of people with latent TB. We do not model specific preventive interventions, but use baseline values from literature (*ϕ* = 0.05, and *ω* = 0.0039 [15]) and estimate how much these parameters would need to reduce to meet the WHO 2035 End TB targets.

### Treatment success rate

Treatment success rate is an indicator of the performance of national tuberculosis control programmes. In addition to the obvious benefit to individual patients, successful treatment of people with infectious TB is essential to prevent the further spread of TB bacteria. We modelled treatment success rate (*τ*) as a constant rate applied throughout the infectious period. This rate includes the proportion of people with active TB that are detected and treated, as well as the inverse of the mean composite time elapsed since becoming infectious to being detected, initiating treatment and becoming non-infectious (mean duration of infectiousness). This differs from the classically defined ‘treatment success proportion’ commonly used in TB research, which is the proportion of those notified cases which successfully complete the treatment course. Systematic reviews show that there is some evidence that ACF can improve the early detection of TB and increase the numbers of people treated for TB [16]. As such, we model the impact of ACF as an increase in *τ*.

### Target scenarios

We run the model with the initial conditions (as described above) and the parameter values in Table 1, to reproduce reported country-level trends for TB incidence in Vietnam and Nepal. Incidence declines reported by WHO between 2002 and 2017 for Vietnam and between 2009 and 2017 for Nepal, are assigned to declines in probability of progression (*ϕ*) and reactivation (*ω*), with the value of the treatment success rate (*τ*) assumed to be constant. This value of *τ* and the rates of decline of the selected parameters are estimated as described below. The estimated parameters are then used to run the model forward in time and calculate the required scale up of efforts, if any, in τ required to reduce the reproduction number to a value that is below the transmission threshold (*R*_0_ = 1) by 2035. Any increase in *τ* would not only lead to a reduction in transmission, but also a reduction in overall incidence towards the End TB incidence targets. It is specifically relevant then to quantify any additional efforts required to meet the End TB incidence targets. To investigate this, we consider a second target where in addition to increasing *τ*, we also calculate the required scale up in *ϕ* and *ω*, if any, to meet the End TB incidence targets by 2035.

### Model calibration and intervention scenarios

We fit a total of four parameters: the effective contact rate (*β*), rate of successful treatment (*τ*) and the declines in progression and reactivation represented by the parameters *r*_*ϕ*_ and *r*_*ω*_, respectively, to WHO incidence data for both countries. We assume that *ϕ* and *ω* decline exponentially and adopt a Bayesian Markov Chain Monte Carlo (MCMC) approach to calibrate the model to obtain posterior distributions for the parameters. We assume gaussian priors for each parameter and construct a likelihood based on WHO incidence data from 2002 to 2017 for Vietnam and from 2009 to 2017 for Nepal. The uncertainty around incidence estimates was assumed to be distributed normally and, in the absence of the sampling distribution for the data, the error variance is sampled as a conjugate prior specified by the parameters *σ*_0_ and *ν*_0_ of the inverse gamma distribution where *σ*_0_ is the initial error variance and *ν*_0_ is assumed to be 1 (as larger values limit the samples closer to *σ*_0_ [22]). We initially minimize an error function (comparing model predictions with WHO data) and use these local minima as initial values for the parameters in the MCMC run. We infer a MCMC chain of length 10^5^ and adopt a burn in of 2 × 10^4^ after assessing the Gelman-Rubins-Brooks potential scale reduction factor (psrf) plots of the posterior distributions.

### Estimating intervention impact (IMPACT TB data)

We calculate the average increase in case notification as a result of the programme in the intervention districts and then use this as an input in the transmission model to predict the incidence and the reproduction number should the efforts be extended to country level. We point out that all data were fully anonymized before we accessed them. We incorporate this into the model by proportionately scaling the treatment success rate parameter *τ*. We consider the direct yield of the intervention which can be defined as the number of people identified with TB through efforts of the intervention. The number of cases found through routine case finding and additional number of cases found as a result of ACF in the IMPACT TB implementation districts are shown in Table 2. The increase at district-level is calculated using the formula $\frac{ACF}{PCF}*\frac{District_{pop}}{Country_{pop}}$ where *District*_*pop*_ is the sum of the implementation district populations and *Country*_*pop*_ is the population of the entire country.

**Table 2:** Number of cases found via active case finding (ACF) and passive case finding (PCF) in the IMPACT TB implementation districts in Nepal and Vietnam.

Country	District	ACF+PCF	PCF	Population size
Vietnam	01–06	533	463	264684
	03–08	889	812	432853
	05 –BC	840	744	685979
	04–12	899	837	569522
	07-HM	798	729	458338
	08-TB	582	520	476645
Nepal	Chitwan	1071	841	678079
	Dhanusa	826	650	828401
	Mahottari	905	743	696650
	Makawanpur	699	657	455554

## Results

### Risk inequality affects the estimation

The model was used to reproduce reported country-level trends for TB incidence in Vietnam and Nepal. Following initialization in 2002 for Vietnam and 2009 for Nepal, the model was fitted to the incidence declines reported by WHO until 2017 using an MCMC approach. The ranges of parameter values that match the data are shown in Table **3**. Posterior parameter distributions change as we vary the variance in individual risk with an increase in the estimated mean values of the contact rate as we increase the variance.

**Table 3:** Estimated parameter distributions (95% credible intervals) for model trajectories in Fig 1.

Country	Parameter	*Var*(*α*) = 5	*Var*(*α*) = 10	*Var*(*α*) = 15
;Vietnam	*rω*	[−0.0136, −0.0134]	[−0.012, −0.0098]	[−0.039, −0.037]
	*rϕ*	[−0.0169, −0.0167]	[−0.017, −0.015]	[−0.0009, −0.0007]
	*τ*	[6.36, 6.40]	[6.38, 6.40]	[5.67, 5.71]
	*β*	[14.7, 15.1]	[10.4, 10.5]	[7.51, 7.61]
Nepal	*r*_*ω*_	[−0.0049, −0.0047]	[−0.004, −0.0038]	[−0.0044, −0.0042]
	*r*_*ϕ*_	[−0.0074, −0.0070]	[−0.0064, −0.006]	[−0.0052, −0.0051]
	*τ*	[5.24, 5.28]	[4.34, 4.40]	[2.68, 2.70]
	*β*	[11.58, 11.66]	[6.76, 6.82]	[3.39, 3.43]

### Active case finding and reducing transmission

**Fig 1:**
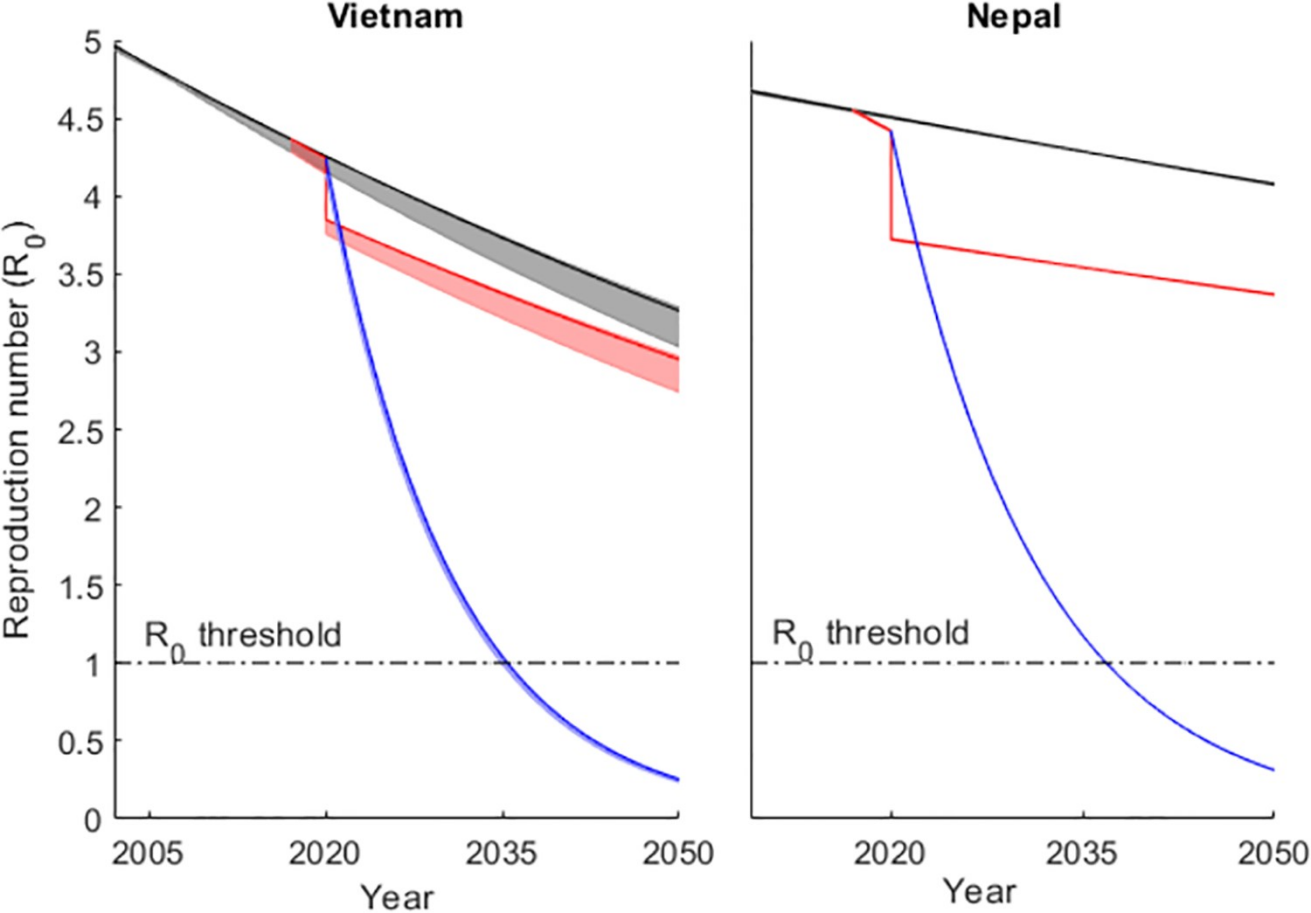
Model projections for the annual reproduction number assuming a risk distribution with variance 10 in Vietnam and Nepal for crossing the transmission threshold R_0_ = 1. The black curves are constructed using the assumption that incidence declines towards 2017 are attributed to reducing disease progression and reactivation with constant rates of decline estimated using an MCMC approach. The shaded areas represent the 95% credible interval of the posterior distribution of the inferred parameters. From 2017, the trajectories split to represent three different scenarios: rates of parameter change are maintained (grey), τ increases according to the increase in case notification in district level (2017:2020), scaled up to country level in 2020 and maintained at this level thereafter (red), a constant rate of increase in τ to reduce R_0_ to 1 by 2035 (blue). Model projections for annual reproduction number in Vietnam and Nepal assuming a risk distribution with variance 5 (S1 fig 1) and variance 15 (S1 fig 4) are provided in the S1 File.

Basic reproduction numbers (*R*_0_) were calculated using countrywide parameter estimates from the fitting procedure above and used for further analysis. First, we prolonged the trajectories until 2050 (grey regions in Fig 1). The resulting trajectories suggest the need for increased efforts to eliminate transmission in both countries by 2035 (timeline used by WHO in the End TB strategy). Results presented in this section are for the intermediate level of risk heterogeneity (variance = 10). Second, we calculated from case numbers in Table 2 how much *τ* increased in study districts over the 3 years of the IMPACT TB project. We estimated instantaneous rates of 0.03 for Vietnam and 0.06 for Nepal which correspond to annual increases in *τ* by 3.4% in Vietnam and 6.6% in Nepal, respectively (small red segments between the beginning of 2017 and the end of 2019). Not surprisingly the effect is small at country level given that IMPACT TB did not have national coverage. To obtain a more meaningful assessment of the potential of this intervention we generated a hypothetical scenario of similar accomplishments throughout the entire countries from 2020 onwards (prolonged red curves). Although the impact in reducing transmission (*R*_0_) would be considerable, this would not be enough to bring the indicator below the threshold *R*_0_ = 1. Third, we set out to estimate how large the rates of increase in *τ* would need to be to ensure that the threshold would be reached assuming a constant rate of increase until 2035. We implement this by considering an instantaneous rate (*r*_*τ*_) at which *τ* increases within the stipulated time $\left(\tau (t)=\tau_{0}e^{r_{\tau }t}\right)$, where *τ*_0_ is the value of *τ* in 2020 (given the increment due to IMPACT TB programme at district level), and *t* is time since the start of intensification of ACF (i.e. time since 2020). This rate *r*_*τ*_ is obtained by direct calculation using the formula for *R*_0_ in Eq 3. The corresponding annual percentage increase in *τ* is 8.9% in Vietnam and 9.9% in Nepal. This suggests that bringing *R*_0_ below 1 by 2035 would require not only annual increases of ACF yield two times higher than those accomplished by IMPACT TB interventions at a national level, but also that the yields would continue to increase at the same rate until 2035.

### Meeting end TB targets

**Fig 2:**
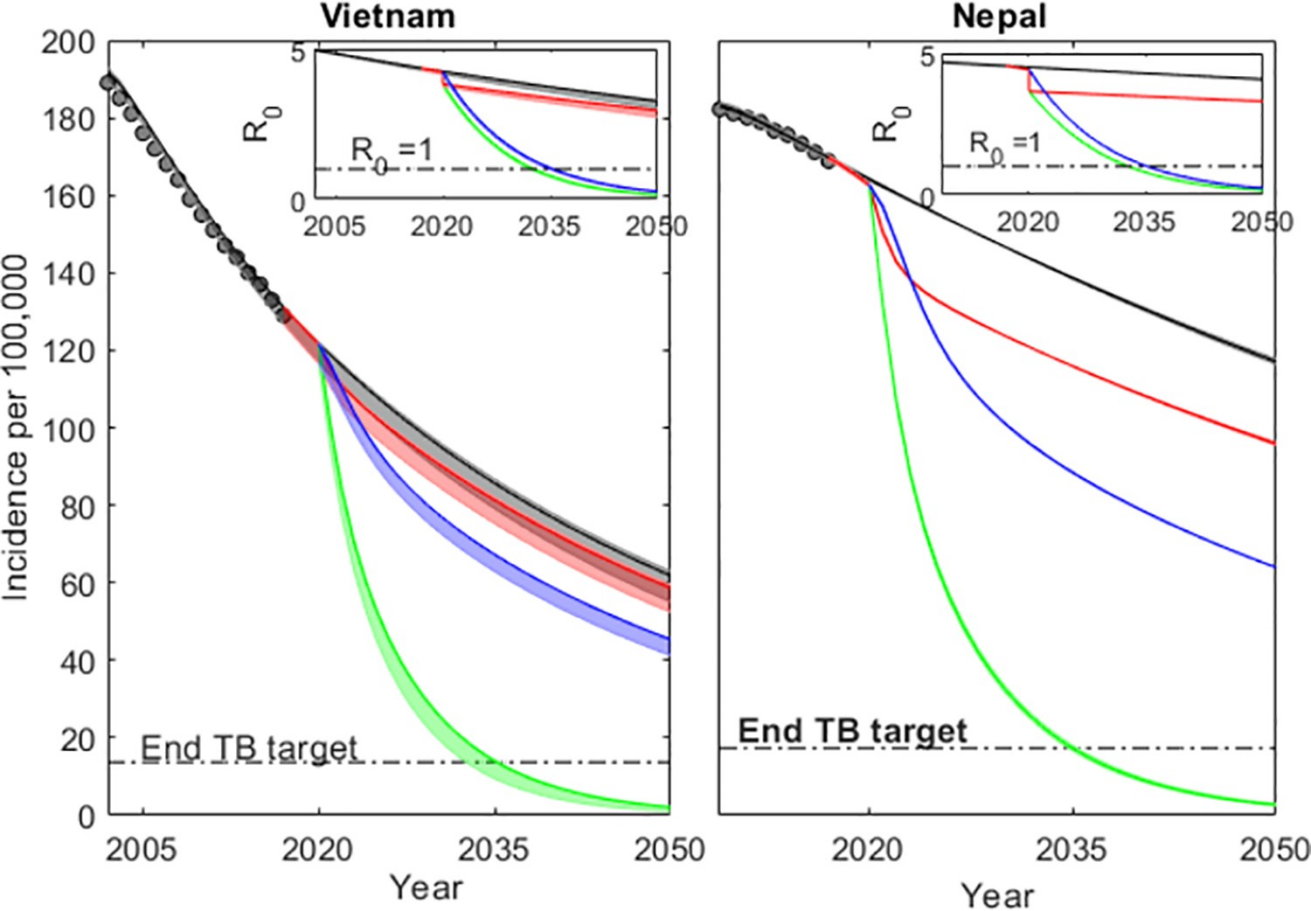
**Model trajectories for annual incidence assuming a risk distribution of variance 10 in both countries: WHO incidence data ((black dots) and model solutions for Vietnam and Nepal.** Incidence decline towards 2017 is attributed to reducing disease progression and reactivation with constant rates of decline estimated using an MCMC approach. Shaded regions were constructed by using a 95% confidence interval of the posterior distribution of the inferred parameters. From 2017, the trajectories split to represent four categories: black, blue and red are the incidence versions of the corresponding colours in Fig 1 and green is an implementation of the required scale up in rates of decline in ϕ and ω by a factor κ required to meet END TB incidence target by 2035 (red) given that R_0_ is reduced to 1. Model trajectories for annual incidence assuming a risk distribution of variance 5 (S1 fig 2) and variance 15 (S1 fig 5) in both countries is provided in the S1 File.

Assuming the ACF strategy applied in IMPACT TB was extended to the national-level (increase in *τ*), the incidence reduces by 6% and 17% in Vietnam and Nepal respectively (red lines in Fig 2). Similarly, if *τ* is scaled to a level that will reduce *R*_0_ to 1 by 2035, there is a steeper decline in incidence in both countries (9.4% and 14.1% in Vietnam and Nepal respectively). However, these declines are not yet fast enough to meet the End TB incidence targets (Fig 2). This indicates the need for a scale up in other parameters in order to meet the End TB Strategy targets, because even a dramatic increase in ACF efforts alone would not suffice. In addition to a scale up in *τ* to reduce *R*_0_ to 1 by 2035, we introduce a scale-up parameter *κ* to account for increased reductions in the rates of progression and reactivation from 2020 onwards. We estimate the value of *κ* required to meet the End TB Strategy incidence rate reduction target, using a binary search approach. The green regions in Fig 2 represent this scenario. The values of *κ* (shown in Table **4**, scenario 2) are substantially different across the different assumptions regarding the magnitude of risk heterogeneity, for each country with the scale up being more effective when the initial decline (pre-scale-up) is predominantly attributed to reducing reactivation as earlier observed in [15].

**Table 4:** Required increase/scale-up of control efforts to meet the targets; scenario 1: Yearly percentage increase in τ to reduce R_0_ to 1 by 2035 and scenario 2: Scale up in decline rates of ϕ and ω to meet the End TB incidence targets given that R_0_ is also reduced to 1 for model trajectories in Fig 1.

	Vietnam		Nepal	
Variance	Scenario 1	Scenario 2	Scenario1	Scenario 2
*Var*(*α*) = 5	[6.4 6.6] %	[8.5 8.9]	[7.9 8.1] %	[24.1 25]
*Var*(*α*) = 10	[8.7 8.9] %	[9.2 11.6]	[9.9 10.1] %	[27.7 29.1]
*Var*(*α*) = 15	[6.1 6.6] %	[4.6 5.2]	[11.1 11.3] %	[22.9 24.1]

These values were estimated using the 95% credible intervals of the posterior parameter distributions.

### Number of cases averted

**Fig 3:**
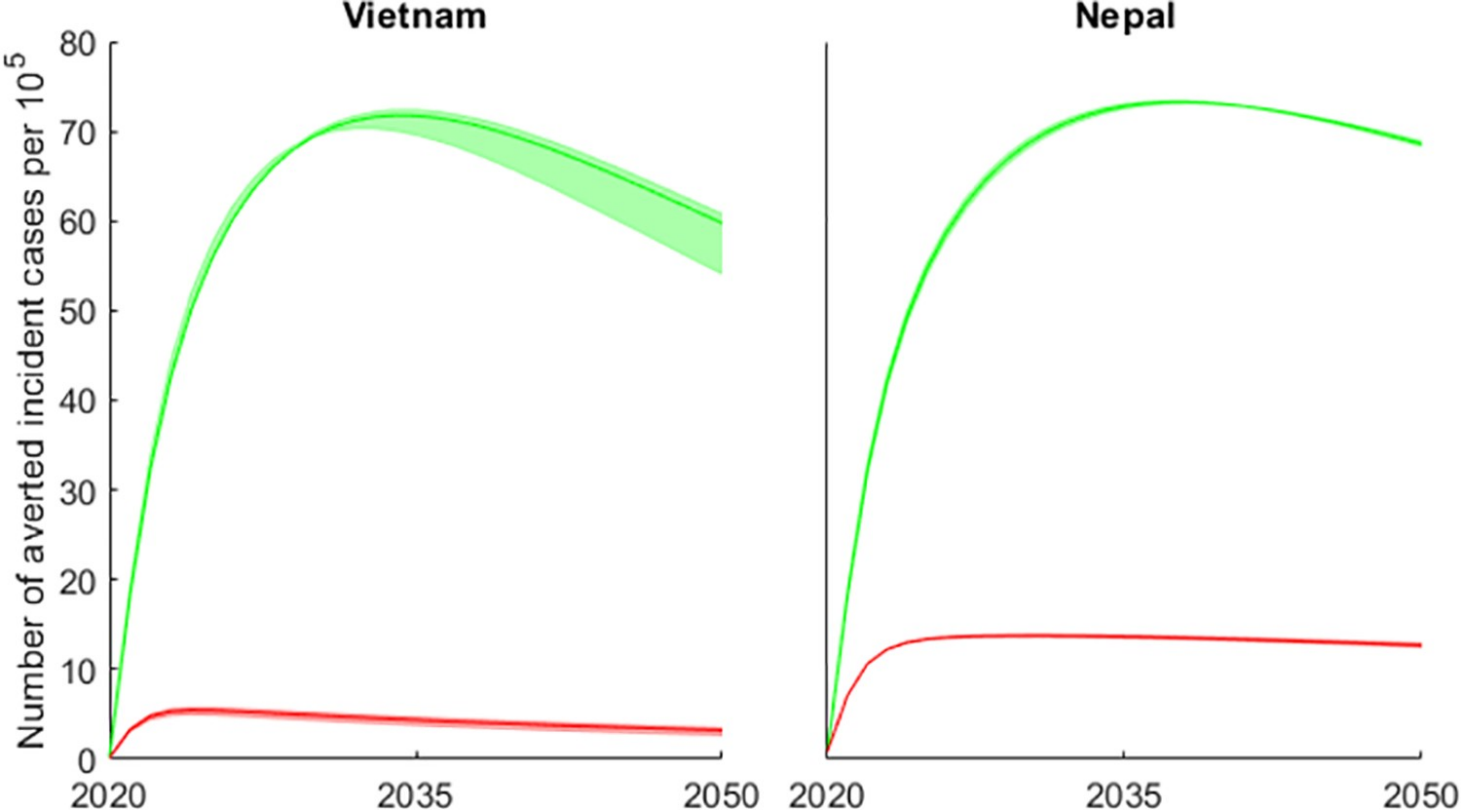
**The projected annual number of tuberculosis cases averted in Vietnam and Nepal between 2020 − 2050 given that preventive interventions are scaled up to meet the End TB incidence targets (green) or that ACF is extended to country level in 2020 (red) and assuming variance of 10.** Similar plots for both countries assuming a variance of 5 (S1 fig 3) and variance of 15 (S1 fig 6) are provided in the S1 File.

We measure the effect of the interventions as the cumulative number of TB cases averted during the intervention relative to the baseline case without the intervention. In the absence of a scale up in preventive interventions (reduction in reactivation and progression) or ACF activities, we project 5,545 (95% credible interval (CI) 5,311–5,562) and 5,618 (95% (CI) 5,602–5,622) incident TB cases per 100,000 in Vietnam and Nepal respectively between 2020 and 2035. Fig 3 shows the annual number of averted TB cases per 100,000 population given that: ACF is expanded to interrupt transmission (reduce *R*_0_ to 1) by 2035 and reductions in *ϕ* and *ω* are scaled up to meet the End TB 2035 incidence targets in Vietnam and Nepal (green); or that active case finding is scaled up to country level and maintained (red). In both countries, the annual number of averted cases per 100,000 as a result of preventive interventions steeply increases until it reaches a plateau in 2035, after which it begins to decline. The predicted cumulative number of cases averted between 2020 and 2035 as a result of preventative interventions would be 1,879 (95% (CI) 1,818–1,896) and 2,926 (95% (CI) 2,915–2,936) in Vietnam and Nepal respectively. On the other hand, we predict that the number of averted cases per 100,000 as a result of a scale up of ACF activities following the IMPACT TB modality alone to country level is 128 (95% (CI) 114–137) and 589 (95% (CI) 583–594) in Vietnam and Nepal respectively.

### Reducing risk

**Fig 4:**
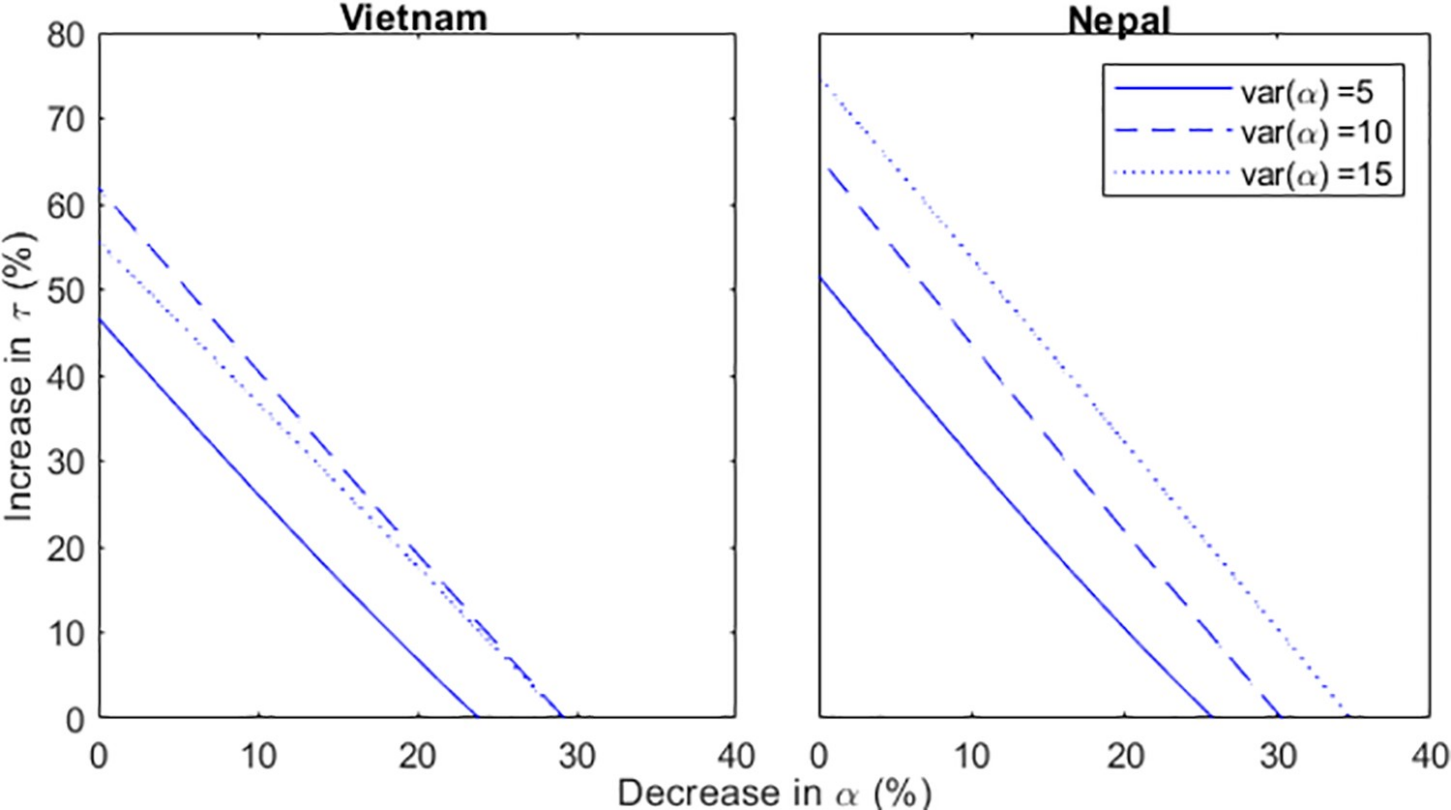
Percentage increase required in τ to reduce R_0_ to 1 between 2017 and 2020 for Vietnam and Nepal.

It has been established that aside from interrupting transmission through early detection, additional interventions to reduce risk of TB are required. This may include activities or policies that aim to reduce exposure and susceptibility to infection in high-risk groups. For each reduced value of *α*_2_ (high risk in relation to mean), we calculate the corresponding value of the increase in *τ* required reduce *R*_0_ to 1 by 2020. Reducing the value of *α*_2_ leads to less required increase in τ to reduce *R*_0_ to 1 (Fig 4), and this is more prominent for higher risk variances.

## Conclusions

The global End TB Strategy aims to reduce global TB incidence to less than 10 per 100,000 by 2035. This is the level observed in most high-income countries today. This is a laudable and long overdue commitment by the global community. However, this target is extremely ambitious and will require a paradigm shift in our approach to TB interventions. ACF has the potential benefit of not only increasing the number of people diagnosed and treated for TB, but also diagnosing TB earlier in the disease course. Ultimately these benefits will translate into reducing TB transmission. Using WHO incidence data, the models presented here estimate the potential impact that scaling up the active case finding strategy applied in IMPACT TB would have on reducing transmission and incidence. We implement this by using the treatment success rate, a parameter in our model that depicts the proportion of cases found and duration of infectiousness. Using programmatic data, we estimate how much this parameter has increased as a result of the implementation of an ACF program, IMPACT TB, in some districts of Vietnam and Nepal. We incorporate this into our model, scale it up to country level and calculate how much increased effort in terms of number of cases found is needed if any, in order to interrupt transmission i.e. reduce the value of *R*_0_ to 1 by 2035. We also calculate the degree to which preventive interventions would need to be scaled up in order to meet the End TB incidence targets. Our results suggest that the amount of effort required in order to reach this goal depends on both the degree of inequality and the driving forces of TB decline in the pre-intervention era.

Our study constitutes a step towards better understanding the effect of ACF on transmission. A major benefit of ACF is that it not only finds cases of TB, but potentially finds these cases much earlier thus reducing transmission and the long-term trajectory of TB in the population [23]. An ACF programme could in principle reduce *R*_0_ below the threshold by 2035, but this would have to be 2–3 times more effective than IMPACT TB in the first year and continue to increase effectiveness at a constant rate until 2035. This suggests that ACF campaigns would have more impact if they are continually scaled over a long period of time. This would not only mean an increase in population coverage, but also a continuous increase in the number of the cases detected. We have also shown that although ACF has a notable effect on reducing the burden of TB transmission within the population, it however does not have the same level of effect on reducing disease incidence. As such, meeting the goal of eliminating TB in high burden countries would require a combination of interventions that tackle different aspects of the infection history. These results may be conservative in the sense that ACF is assumed to detect random cases in the population. There is, however, evidence that IMPACT TB has targeted people at higher risk than average [24]. Accounting for this would result in higher effectiveness estimates.

This study underscores the importance of implementing a complementary package if interventions at national level, which address multiple rivers of the epidemic, rather than isolated TB interventions which are unable to have significant impact on national epidemics when implemented individually. We have shown that reducing the risk of TB among vulnerable population reduces the degree of effort required for other interventions to be effective in achieving reduction of transmission such that *R*_0_ is below 1. Here, by reducing risk we do not mean targeting ACF efforts towards high risk group individuals, but instead applying interventions that aim at improving the social, economic, and environmental determinants of individuals in the high-risk groups. These interventions essentially are geared towards TB prevention, care and support and could be TB specific, TB inclusive, or TB sensitive depending on the target population [25]. Evidence of the impact of these interventions is gradually being gathered [26,27]. In essence, if current ACF strategies are complemented with efforts to address TB risk factors and social determinants, then it would be more effective.

We do not model specific preventive interventions but use baseline values of progression and reactivation rates from literature and estimate how much reduction is required in order to meet the End TB 2035 incidence targets. Our results suggest that to meet the goal in addition to a scale up in ACF activities, reactivation rates would have to reduce to 0.0006 and 0.0005 in Vietnam and Nepal respectively. These reductions of over 80% would lead to reactivation rates lower than what is observed in developed countries where the burden of TB is low [28,29]. Although TB preventive therapy (TPT) reduces the risk of tuberculosis by 60% at the individual level [3,30], attaining such low levels of reactivation on a population level is probably not achievable.

We project that within 15 years (between 2020 and 2035), a combination of country scaled ACF and secondary preventive interventions could avert more than half of incident TB in Vietnam and Nepal. Our projections show that much of the effect of scaling up preventive interventions (in terms of number of cases averted) accrues in the first few years after implementation. The diminishing effect over time suggests a saturation effect, which might imply that such interventions could be used within an adaptive control strategy. We expect that the scaling up of preventive interventions in settings with a low TB burden would be less effective, nonetheless, our results suggest that efforts to both prevent and rapidly detect and treat infectious individuals will produce important health benefits.

As with any modelling study, there are limitations to take note of. First, we have used WHO incidence estimates, which are themselves derived from certain assumptions. Nonetheless, our findings should be seen as an illustration of principle which could be refined with improved TB burden estimates such as a series of prevalence surveys. Second, there is no reason for identical scale-up factors for the two preventive processes (reduction in progression and reactivation) given that a scale up tends to be more effective when the incidence decline pre-scale up is predominantly governed by reduction in reactivation (as determined by the parameter estimation procedure). Third, the IMPACT TB project was implemented at district-level. As such, scaling the achieved impact to a national level represents a best-case scenario. Inefficiencies and reduction in yields are invariably seen when public health interventions are scaled-up to a national level for complex multi-factorial reasons. The aim of this work however is to provide an estimate of the level of effort needed, and the contribution of different types of intervention, to meet the goal of pushing TB transmission below the threshold in Nepal and Vietnam. Further work that makes use of district level data from the implementation districts would reflect more accurately the impact of ACF in these districts. Fourth, studies have shown that a proportion of TB cases identified during ACF (measured by direct yield) would have found their way to treatment through the passive system eventually in the absence of the ACF intervention. However, the cases would have most likely been found at a later disease stage allowing them more time to transmit. A more accurate measurement of the impact of ACF would therefore include a measurement of the duration of infectiousness, although this is not possible to measure directly and surrogate markers such as duration of symptoms are subject to strong recall bias.

In conclusion, evaluating the impact of ACF programmes based on TB incidence alone is unlikely to reflect their true benefit and sustaining these programmes can have an important impact on reducing TB transmission in the population. We have shown that dramatic intensification of TB efforts is needed to bring *R*_0_ for TB below 1, and to achieve the End TB Strategy goals, efforts must be comprehensive and sustained to be effective. The findings from this study should motivate funding bodies, implementers and policy makers to take steps towards having a holistic and unrelenting approach towards achieving TB elimination.

## Supporting information

S1 File Contains all the supporting tables and figures. (DOCX)
