# Supplementary material for: The impact of active case finding on transmission dynamics of tuberculosis: A modelling study
Source: PLoS One. 2021 Nov 19;16(11):e0257242. doi: 10.1371/journal.pone.0257242 (PMC8604297; doi:10.1371/journal.pone.0257242)
Supplement: S1 File — (DOCX) [file pone.0257242.s001.docx]

**Supplementary Information**


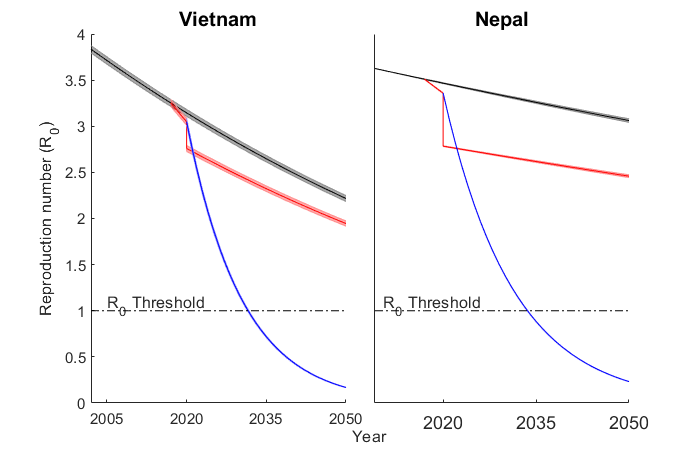


**S1 fig 1**: *Model projections for the annual reproduction number assuming a risk distribution with* $variance = 5$ *in Vietnam and Nepal for crossing the transmission threshold* $R_{0}=1$*. The black curves are constructed using the assumption that incidence declines towards 2017 is attributed to reducing disease progression and reactivation with constant rates of decline estimated using an MCMC approach. The shaded areas represent the* $95\%$ *credible interval of the posterior distribution of the inferred parameters. From 2017, the trajectories split to represent three different scenarios: rates of parameter change are maintained (grey), τ increases according to the increase in case notification in district level (2017:2020) and scaled up to country level in 2020 and maintained at this level thereafter (red), a constant rate of increase in τ to reduce* $R_{0}$ *to 1 by 2035 (blue): the rate of increase is obtained using direct calculation.*


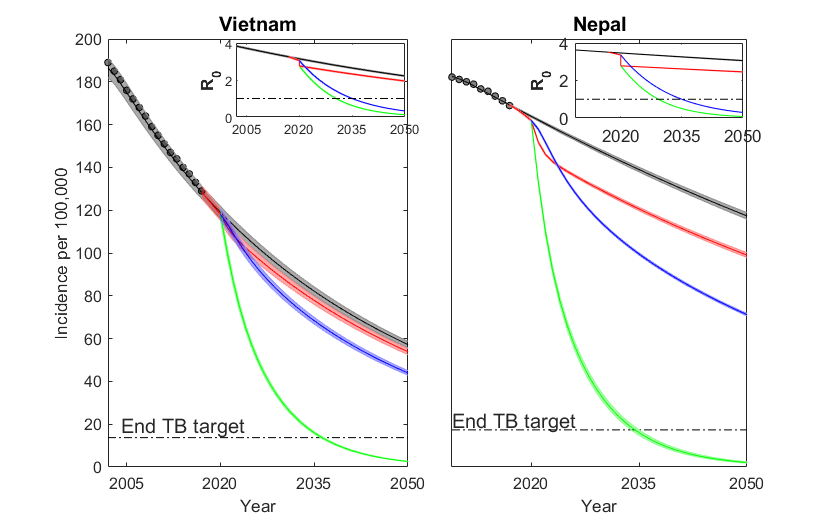


**S1 fig 2**: *Model trajectories for annual incidence assuming a risk distribution of* $variance=5$ *in both countries: WHO incidence data ((black dots) and model solutions for Vietnam (a) and Nepal (b). Incidence decline regions towards 2017 is attributed to reducing disease progression and reactivation with constant rates of decline estimated using an MCMC approach. These regions were constructed by using a 95% credible interval of the posterior distribution of the inferred parameters. From 2017, the trajectories split to represent four categories: the black, blue, and red curves are the corresponding incidence curves of S1 fig and the green curve is an implementation of the required scale up in rates of decline in ɸ and ω by a factor κ required to meet END TB incidence target by 2035 (red) given that* $R_{0}$ *is reduced to 1.*


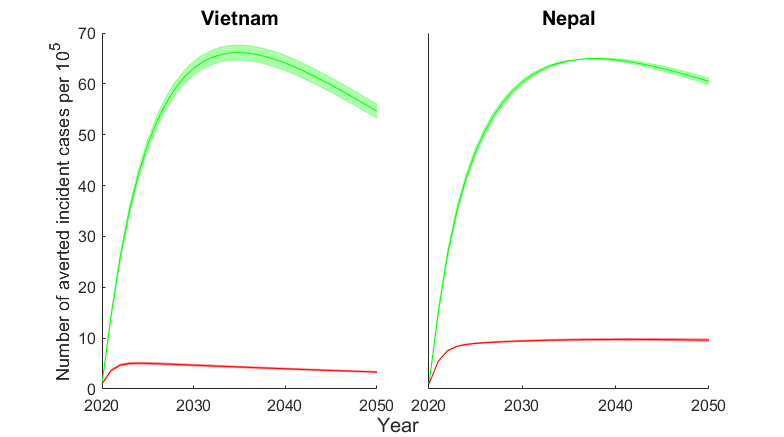


**S1 fig 3**: *The projected annual number of tuberculosis cases averted in Vietnam and Nepal between 2020 − 2050 given that preventive interventions are scaled up to meet the End TB incidence targets (green) or that ACF is extended to country level in 2020 (red). The variance of the risk distribution in the simulations is 5.*


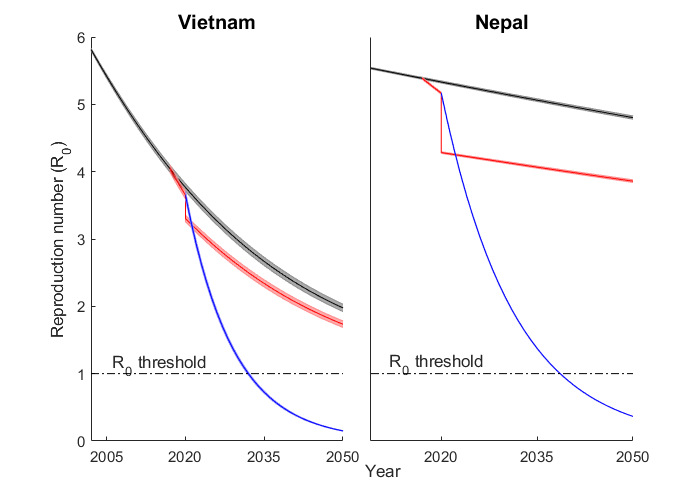


**S1 fig 4**: *Model projections for the annual reproduction number assuming a risk distribution with* $variance = 15$ *in Vietnam and Nepal for crossing the transmission threshold* $R_{0}=1$*. The black curves are constructed using the assumption that incidence declines towards 2017 is attributed to reducing disease progression and reactivation with constant rates of decline estimated using an MCMC approach. The shaded areas represent the* $95\%$ *credible interval of the posterior distribution of the inferred parameters. From 2017, the trajectories split to represent three different scenarios: rates of parameter change are maintained (grey), τ increases according to the increase in case notification in district level (2017:2020) and scaled up to country level in 2020 and maintained at this level thereafter (red), a constant rate of increase in τ to reduce* $R_{0}$ *to 1 by 2035 (blue): the rate of increase is obtained using direct calculation.*


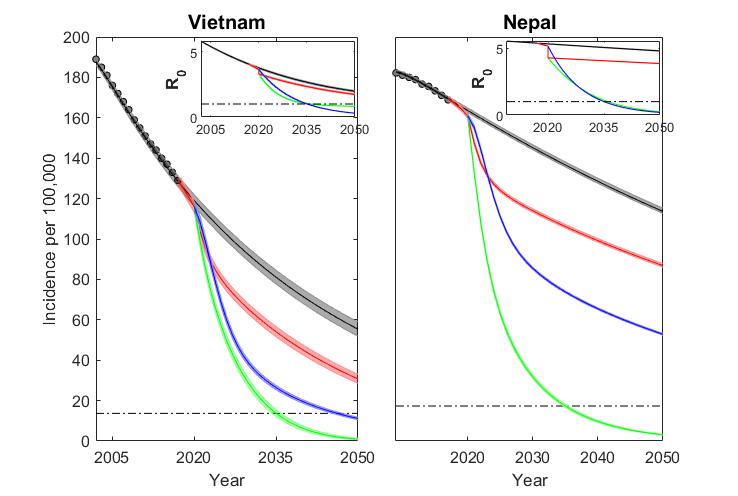


**S1 fig 5**: *Model trajectories for annual incidence assuming a risk distribution of* $variance=15$ *in both countries: WHO incidence data ((black dots) and model solutions for Vietnam (a) and Nepal (b). Incidence decline regions towards 2017 is attributed to reducing disease progression and reactivation with constant rates of decline estimated using an MCMC approach. These regions were constructed by using a 95% credible interval of the posterior distribution of the inferred parameters. From 2017, the trajectories split to represent four categories: the black, blue, and red curves are the corresponding incidence curves of S4 fig and the green curve is an implementation of the required scale up in rates of decline in ɸ and ω by a factor κ required to meet END TB incidence target by 2035 (red) given that* $R_{0}$ *is reduced to 1.*


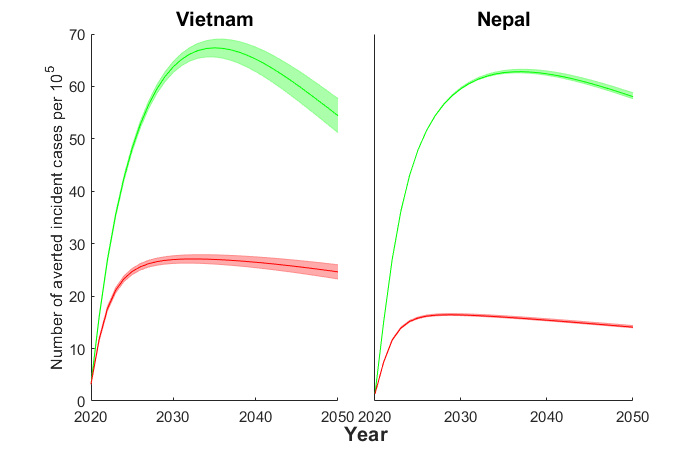


**S1 fig 6**: *The projected annual number of tuberculosis cases averted in Vietnam and Nepal between 2020 − 2050 given that preventive interventions are scaled up to meet the End TB incidence targets (green) or that ACF is extended to country level in 2020 (red). The variance of the risk distribution in the simulations is 15.*
